# Pathways for Diagnosis and Multimodal Management, Including Botulinum Neurotoxin Therapy, in Shoulder Conditions Following Acquired Central Nervous System Lesions

**DOI:** 10.3390/toxins17080385

**Published:** 2025-07-31

**Authors:** Bo Biering-Sørensen, Carlos Cordero-García, Chris Boulias, Damon Hoad, Djamel Bensmail, Franco Molteni, François Genêt, Jörg Wissel, Jorge Jacinto, Philippe Marque, Steffen Berweck

**Affiliations:** 1 Rigshospitalet, Neurological Department, Movement Disorder and Pain Research Center, 2600 Copenhagen, Denmark; 2 Hospital Universitario Juan Ramón Jiménez, 21005 Huelva, Spain; carlos.cordero.sspa@juntadeandalucia.es; 3 West Park Healthcare Centre, Toronto, ON M6M 2J5, Canada; chris.boulias@uhn.ca; 4 Warwick Medical School, University of Warwick, Warwick CV4 7AL, UK; damon.hoad@nhs.net; 5 Raymond-Poincaré Hospital, GH Université Paris Saclay, University of Versailles Saint-Quentin-en-Yvelines, 92380 Garches, France; djamel.bensmail@aphp.fr (D.B.); francois.genet@aphp.fr (F.G.); 6 Villa Beretta Rehabilitation Center, 23845 Costa Masnaga, Italy; franco56.molteni@gmail.com; 7 Neurology and Psychosomatic at Wittenbergplatz, 10787, Berlin and University of Potsdam, 14469 Potsdam, Germany; joerg@schwarz-wissel.de; 8 Centro de Medicina de Reabilitação de Alcoitão, 2649-506 Alcabideche, Portugal; jor.jacinto@netcabo.pt; 9 Centre Hospitalier Universitaire de Purpan, 31059 Toulouse, France; marque.ph@chu-toulouse.fr; 10 Ludwig-Maximilians-University of Munich, 80539 Munich, Germany; steffen.berweck@me.com; 11 Schön Klinik Vogtareuth, 83569 Vogtareuth, Germany

**Keywords:** botulinum toxins, type A, spasticity, pain, shoulder

## Abstract

There is limited published guidance available to help less experienced practitioners assess and manage shoulder conditions, including spasticity, after acquired central nervous system (CNS) lesions. To address this gap, 11 spasticity and dystonia experts convened in a 2023 meeting to build on existing guidance, provide consensus on best treatment practice, and develop expert recommendations to guide the diagnosis and treatment of complications of shoulder conditions following CNS lesions. Presentations by each expert on diagnosis and management were followed by discussion; consensus on assessment and treatment practices was identified and recommendations developed. The expert panel recommended an assessment approach structured using the following components: patient history, including interpretation of reported symptoms; observation of postures and pain responses; clinical examination with targeted tests for specific signs; diagnostic tests; and assessment of upper limb impairment, activity limitations, and participation restrictions. This assessment process and the recommended measures recognize the importance of identifying shoulder involvement in upper limb spasticity as part of the diagnostic process in shoulder conditions following CNS lesions. These recommendations provide a practical approach to diagnosis and treatment for clinicians who are less experienced in evaluating and treating such conditions, simplifying otherwise complicated clinical scenarios.

## 1. Introduction

Normal shoulder function is essential for almost all active movements of the arm and for gait. The primary physiological function of the shoulder is to facilitate movement of the upper limb so that the hand can be correctly positioned and deployed in a multitude of everyday tasks. Thus, abnormal shoulder function adversely affects hand function by constraining upper limb movement [1].

Spasticity commonly complicates management of shoulder conditions following central nervous system (CNS) lesions [2,3,4]. Recent studies have suggested that upper limb spasticity affects around one-quarter to one-third of patients in the 12 months after a first stroke [5,6], with important functional and quality-of-life implications. Spasticity of shoulder conditions following CNS lesions may be associated with pain and deformity, limiting both passive and active movements [7]; indeed, shoulder pain is the most common post-stroke pain disorder [8]. Spasticity and its sequelae can reduce quality of life because of impaired mobility, hygiene, social functioning, and sleep and increased caring needs [4,9,10] and can cause or exacerbate low mood, lack of motivation, and social isolation [11].

Botulinum neurotoxin type A (BoNT-A) is recognized as an important treatment option for patients with spasticity caused by a CNS lesion [12,13,14]. The aims of treatment are to facilitate restoration of active function and improve passive range of movement, relieve pain, reduce involuntary movements, and prevent the development of pathological postures. Clinical studies in patients with shoulder spasticity following CNS lesions have shown improvements in muscle tone (as measured by the Tardieu Scale, the Modified Ashworth Scale [MAS], and the Ashworth Scale shoulder sum score), active and perceived function (measured using the Modified Frenchay Scale and the Disability Assessment Scale, respectively), and pain (assessed using the Spasticity-Associated Arm Pain Scale and the Pain Numeric Rating Scale) after treatment with BoNT-A [15,16,17,18]. However, although evidence shows the benefit of therapeutic goals and treatment related to shoulder spasticity [7,19], the condition and its complications remain under-recognized and undertreated [15,16,17]. This may also apply to children with unilateral cerebral palsy (CP). Although BoNT-A is acknowledged as an efficient adjunct to other evidence-based therapies in such cases [20], impairment of the shoulder muscles in these children may also be under-reported and undertreated [21].

There is limited published guidance on the assessment [22] and management [1,23] of shoulder conditions, including spasticity, following acquired CNS lesions. Expert recommendations have also described BoNT-A injections, clinical evaluation tools, and outcome expectations [1,23]. However, these published recommendations are brief, and no formal guidelines have been produced. Thus, less experienced practitioners may lack confidence in assessing and treating these conditions or may even be unaware of how to approach treatment. To bridge this knowledge gap, experts in the diagnosis and management of spasticity and dystonia following acquired CNS lesions, including stroke, were invited to participate in an expert exchange meeting on the importance of the shoulder, its functional anatomy, and the problems that exist in this patient population. The purpose of the meeting was to reach a consensus on best practice and formulate expert recommendations to guide healthcare professionals involved in diagnosing and treating these patients. This paper summarizes the experts’ discussions and recommendations. Note that these recommendations are based on expert consensus without systematic literature review or empirical validation.

## 2. Results

### 2.1. Diagnosis

#### Clinical Evaluation and Investigation

The experts recommended that all pathological movements and postures, including musculoskeletal impairments, should be assessed and characterized as either hypokinetic or hyperkinetic. Hypokinetic movement patterns accompanied by flaccid paresis and (for example) subluxated shoulder should be identified, and further diagnostic work-up (e.g., electromyography [EMG], X-ray, ultrasound [US]) should be performed to guide selection of an appropriate treatment pathway, which may include shoulder support or neuromuscular electrical stimulation. Hyperkinetic movement patterns (e.g., phasic or tonic involuntary activation; velocity-dependent increase in muscle tone; muscle co-contraction during activation; spastic dystonia and associated reactions; dyssynergia) should be assessed, and those that are disabling for patients should be treated with, for example, multi-focal or multi-segmental BoNT injection depending on the distribution of muscle overactivity.

It is important to note that hypokinetic movement patterns typically occur immediately after a CNS lesion, whereas the development of hyperkinetic movement patterns is usually delayed. However, according to expert consensus, both types of pathological movement pattern may evolve over time because of treatment effects or pattern modification, regardless of their time of onset. Movement patterns also differ according to etiology (e.g., multiple sclerosis vs. traumatic brain injury vs. spinal cord injury). A systematic approach to examination and ordering investigations should consider all neural and musculoskeletal pathologies affecting the shoulder joint. Practitioners should also consider joint axes (planes), degrees of freedom, range of motion, and stability. Non-invasive diagnostic tools (e.g., X-ray, US, EMG) can be useful for delineating neural and musculoskeletal pathologies and can be used before specific treatments. Diagnostic nerve blocks can also be used to identify contributing pathologies. The Victoria Verona (ViVe) algorithm is an established tool that guides the use of targeted nerve blocks to aid the differential diagnosis of new-onset shoulder conditions following CNS lesions [8].

**Figure 1:**
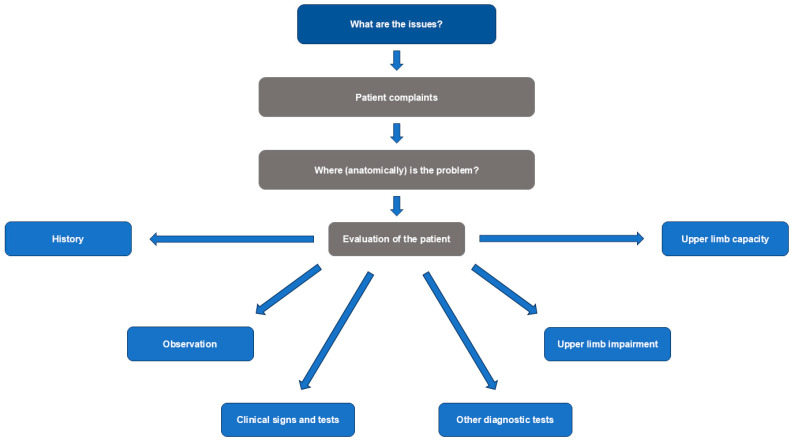
Clinical evaluation and investigation based on a functional anatomical approach.

During the workshop on clinical examination and investigation, the experts noted four main components in the evaluation of patients with a shoulder condition following acquired CNS lesions: patient history, including interpretation of reported symptoms; observation of postures and pain responses; clinical examination with targeted tests for specific signs; and diagnostic tests (Table 1). However, not all assessments should be used for every patient, and some tools will not be available in every clinic. The experts also created a comprehensive list of clinical and instrumental evaluations within each of these categories for assessing patients with suspected disabling shoulder spasticity (Table 1), based on the functional anatomical approach shown in Figure 1.

The first step in evaluation is to classify and characterize the presenting symptoms. The expert panel formulated the following questions to aid this process:

- What is the impact on passive and active function, including posture at rest and in gait?
- Is there pain with passive or active movement, or at rest?
- What are the neural and musculoskeletal contributing factors?
- Is the etiology neurological or non-neurological?
- For neurological causes, what are the patterns of pathological movement and posture?
- Is the movement pattern hypo- or hyperkinetic?
  - If it is hyperkinetic, is it tonic or phasic?

Presenting symptoms must be assessed with reference to posture, involuntary movements, pain, and activity limitations, and the precise anatomical location of the problem should be determined. This may be the joint, capsule, ligament, bursa, tendon, muscle, or nerve.

Experts also emphasized that shoulder conditions following CNS lesions must be assessed within the context of the whole biomechanical system, including the trunk, proximal and distal joints, and gait. Tools other than nerve blocks may be needed in the chronic phase (e.g., in patients with osteoarthritis or rotator cuff lesions), especially when patients have comorbid musculoskeletal or neural degenerative diseases in addition to spastic movement disorders or postures. In these cases, information from other diagnostic tools, such as imaging or neurophysiology, can aid the interpretation of clinical examinations and diagnostic nerve blocks.

### 2.2. Management Strategies

#### 2.2.1. Pain

The expert panel noted that practitioners should be aware that pain arising from shoulder conditions following CNS lesions may have a multifactorial etiology, and there may be biomechanical drivers of nociceptive pain (e.g., subluxation, shoulder impingement, subacromial bursitis, capsulitis, rotator cuff tears, glenohumeral osteoarthritis). Clinical analysis can be complicated by interactions between pathologies; for example, capsulitis may worsen spasticity.

Characterizing pain, even at a basic level (e.g., whether it occurs at rest or on movement), is important. The experts agreed that an algorithm is needed because the treatment approach depends on the etiology of the pain; it is important to differentiate between neuropathic, nociceptive, and nociplastic pain to guide treatment, although this can be challenging. Both neuropathic and stretch-related pain are modulated by BoNT-A [29,30], so treatment with BoNT-A does not help identify the type of pain. However, based on their own personal experience, published data from a systematic review and meta-analysis [31], a randomized controlled trial [32], and the TOWER study [17], the experts highlighted BoNT-A as an important tool for managing shoulder pain associated with spasticity after a CNS lesion. The panel also acknowledged that suprascapular nerve block can play a complementary role in pain management, as it provides analgesia for several weeks and acts as an adjunct to BoNT-A treatment [33]. In patients presenting with central neuropathic pain, which can worsen nociceptive pain (and vice versa), the experts also advised following the recent treatment recommendations from the Special Interest Group on Neuropathic Pain (NeuPSIG) of the International Association for the Study of Pain (IASP) [34].

#### 2.2.2. Multimodal Treatment

**Figure 2:**
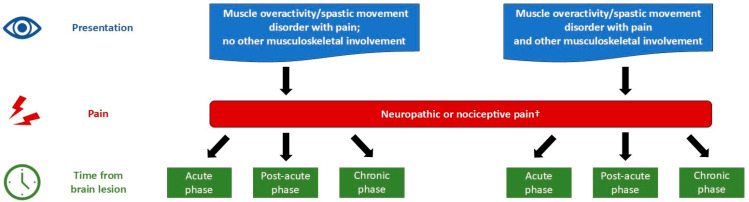
Recommended categorization of patients for management of shoulder conditions following acquired central nervous system lesions. ^†^ Oncoplastic pain is excluded. Nociceptive pain may be classified as during rest, sleep, activity, passive movement, or a specific task.

Figure 2 presents the experts’ recommendations for categorizing patients with acquired CNS lesions and painful shoulder spasticity. Six patient subgroups are described, defined by the presence or absence of musculoskeletal involvement (excluding muscle overactivity) and time since lesion (acute, post-acute, and chronic phases).

Treatment recommendations for each subgroup are provided in Table 2. Although this article provides guidance for physicians, spasticity management should involve a multidisciplinary team [35], including therapists, whose assessments provide valuable input for treatment decisions [36].

Patients in the acute phase should undergo assessment and diagnosis, using the approach described in Figure 1 and Table 1, before treatment decisions are made. For patients in the post-acute and chronic phases, previous treatment should be reviewed to determine what has worked, what needs to continue or stop, and what needs to be started.

The expert panel observed that reducing hypertonia is not always the primary objective of management in patients with shoulder spasticity. Thus, treatment options other than those shown in Table 2 may be indicated. These may include corticosteroids, non-steroidal anti-inflammatory drugs, and visco-supplementation with hyaluronic acid and platelet-rich plasma. There are few neuro-orthopedic surgical procedures for neurological shoulder conditions, although tenorrhaphy of the biceps brachii (caput longum) may be considered in some individuals.

In adults and children with unilateral CP, BoNT-A is an effective therapy when combined with other treatment modalities, especially occupational therapy [20]. In many patients, it cannot be expected that a paretic hand will regain its full potential; after stroke, the concept of using an ‘assisting hand’ is helpful in accomplishing everyday tasks since most of these tasks require a bimanual strategy. This concept is used from the very beginning of development and treatment in children with unilateral CP [39,40]. It may also be a valid principle for adults after a stroke, and a measure to capture the performance of the ‘assisting hand’ has been validated in these patients [41]. Beyond therapies that aim for improvement in motor competence, strategies are needed that make the most of patients’ cognitive abilities and help them to perform activities of daily living [42].

#### 2.2.3. Goal Setting

Goals should be patient centric, developed in collaboration with the patient and the patient’s family [35,37], and used to guide treatment selection with a focus on active goal setting [35]. Several tools are available to facilitate goal setting and achievement in shoulder spasticity, including the GAS-eous tool and the Goal-Oriented Facilitated Approach to Spasticity Treatment (https://go-fast.toxnet.net; accessed on 21 May 2025) [43].

Most frequently, the objectives of managing shoulder and proximal upper limb spasticity using BoNT and concurrent therapies are to relieve pain; facilitate dressing, hygiene, and limb positioning; improve sleep quality and walking comfort; and prevent the worsening of contractures [1,23,44]. Additionally, as shoulder dysfunction following an acquired CNS lesion can affect posture and body image, potentially reducing patients’ self-esteem and social confidence [1], improved body image perception can be selected as a goal in the GAS-eous tool [45]. Notably, it is one of the most frequently achieved goals in pooled analyses of upper limb spasticity studies [46,47].

Two studies—the phase IV, real-world ULIS-II study and the open-label, phase III AUL study—demonstrated that pain relief was a more frequent primary goal among patients receiving BoNT-A in shoulder muscles than among those not receiving shoulder treatment [48]. The ULIS-II study comprised one treatment cycle and did not characterize the etiology of pain. The study evaluated patients in the chronic phase (4–7 years post-stroke), who likely had many complications in addition to spasticity.

#### 2.2.4. Case Study

During the meeting, experts discussed several real-life clinical cases to demonstrate how they assessed shoulder conditions, the management strategies used, and outcomes of treatment. One representative case report is described here.

A 78-year-old male with a total anterior circulation infarct was treated with mechanical thrombectomy in October 2021. At 6 weeks post-infarct, the patient developed three spastic patterns involving the shoulder, flexed elbow, and a clenched fist that gave rise to a non-functional hand. The shoulder was internally rotated and adducted, although assessment of spasticity using the MAS was not possible because of pain that limited passive movement. Additionally, the patient had a MAS score of 2 for elbow flexors and finger flexors and a visual analog scale (VAS) score of 10 for spastic shoulder pain at flexion, abduction, and external rotation; pain was experienced with any kind of movement. In addition to spasticity, the patient had a partial supraspinatus tear and subluxation of the glenohumeral joint.

The initial treatment approach was BoNT-A (incobotulinumtoxinA), injected into the pectoralis major (80 U), subscapularis (70 U), latissimus dorsi (50 U), biceps brachii (50 U), brachialis (50 U), flexor digitorum superficialis (40 U), and flexor digitorum profundus (40 U) muscles. The total dose of BoNT-A was 380 U. The patient also received suprascapular nerve block, axillary nerve block, pectoralis I nerve block, physical therapy, and baclofen 10 mg every 8 h; the latter was included because it had been administered previously, and an axillary nerve block was performed to relieve the patient’s shoulder pain, since articular branches of the axillary nerve are involved in the sensory innervation of the anterior and posterior shoulder capsule. Physical management of pain included stretching and joint stabilization techniques.

The treatment goals for the first cycle of BoNT-A were to reduce pain (VAS score ≤ 4) at 8 weeks post-injection; to facilitate axillary hygiene 4 weeks post-injection; to facilitate caregiver assistance in dressing tasks 4 weeks post-injection; and to prevent deformities/contractures and reduce muscle tone. All goals were achieved except for a reduction in shoulder pain: the VAS score was 5 at the pre-specified time point.

The patient received an increased total dose of incobotulinumtoxinA (500 U) in the second cycle (pectoralis major, 110 U; subscapularis, 80 U; latissimus dorsi, 60 U; biceps brachii, 80 U; brachialis, 80 U; flexor digitorum superficialis, 50 U; and flexor digitorum profundus, 50 U). Additional therapies administered were pulsed radiofrequency suprascapular and axillary nerve treatment, physical therapy, and baclofen 10 mg every 8 h.

Treatment goals for the second cycle were the same as for the first cycle. All goals were achieved, with a VAS score of 2 for shoulder pain at 8 weeks post-injection.

## 3. Discussion

Upper limb spasticity is a common consequence of CNS lesions [5,6], often causing pain and impairing movement, function, and quality of life [4,7,8,9,10]. As shoulder function is essential for gait and active arm movements, shoulder involvement should always be considered when upper limb spasticity presents after CNS lesions.

However, shoulder spasticity and its complications are under-recognized and undertreated in clinical settings [15,16,17], and published guidance on their management remains scarce [1,22,23]. Hoad et al. [22] recently made important progress towards addressing this gap by offering an introductory set of practical guidelines, developed by six spasticity experts, to help less experienced injectors identify and assess shoulder involvement in patients with arm spasticity. Those recommendations focus primarily on identification and assessment, providing a structured, systematic approach organized around the ‘three Ps’ (Posture, Purposeful activity, Pain) framework [22]. With the same goal of improving confidence in shoulder spasticity management among less experienced physicians, and similarly emphasizing the importance of differential diagnosis, the current article extends this groundwork by providing detailed treatment guidelines that address evolving pathologic movement patterns and the complex, multifactorial pathologies underlying shoulder dysfunction in patients with CNS lesions.

Diagnosing shoulder involvement in upper limb spasticity after CNS lesions is clinically challenging due to evolving presentations, differing etiologies, and the complex interplay between neurologic and musculoskeletal factors, including soft tissue contractures. In addition, many individuals affected by neurologic injuries—especially stroke—are middle-aged or older [49], and these age groups have a high prevalence of pre-existing musculoskeletal shoulder pathologies [50,51]. This can further complicate the identification of shoulder spasticity following a CNS lesion. Evaluating shoulder dysfunction in these patients requires a thorough, systematic approach that considers both neurologic and musculoskeletal pathologies, as well as joint mechanics. Non-invasive tools such as X-ray, US, and EMG can help identify underlying pathologies and guide treatment decisions. US also plays a vital role in enabling accurate placement of BoNT-A injections, enhancing precision and safety. US can also minimize complications associated with blind injections [52]. Targeted diagnostic nerve blocks, guided by the ViVe algorithm, can also aid differential diagnosis in new-onset shoulder conditions. However, although the ViVe algorithm is an established and helpful tool, several limitations should be noted. For example, administering the lateral pectoral nerve block according to the ViVe algorithm may not be diagnostically effective in all patients due to anatomical variability in the lateral nerve [53] and the algorithm’s omission of the medial pectoral nerve block, which targets the sternocostal region of the pectoralis major [54]—a key contributor to common patterns of shoulder spasticity. In addition, nerve blocks are invasive, and more than one (i.e., axillary, lateral pectoral) may be needed to address shoulder pain. Furthermore, the ViVe algorithm does not account for the evaluation of motor control or for conditions such as rotator cuff lesions, tendonitis, subluxation of the glenohumeral and acromioclavicular joints, and glenohumeral osteoarthritis.

The expert panel recommended a structured approach for the evaluation and differential diagnosis of shoulder conditions in this patient population, centered on four key components: patient history, including interpretation of reported symptoms; observation of postures and pain responses; clinical examination with targeted tests for specific signs; and diagnostic tests (Table 1). This structured approach not only facilitates the identification of shoulder involvement in upper limb spasticity and the delineation of underlying neural and musculoskeletal pathologies but also supports goal-oriented care by enabling clinicians to tailor treatments to individual patient needs. The goal of such tailored interventions, guided by this comprehensive assessment approach, are to reduce pain, support functional independence in daily activities, enhance participation, and prevent further musculoskeletal complications.

Pain is common in patients with shoulder conditions following CNS lesions [55,56]. For example, shoulder pain occurs in 17% of patients with stroke at week 1 and in 30–70% of patients with stroke overall and may peak at up to 4 months [8]. Pain can limit both shoulder function and patient engagement with upper limb rehabilitation, which leads to limb disuse and indirectly accelerates the development of contracture. One-quarter (25%) of adults presenting to hospital with stroke develop a shoulder contracture within 6 months, and this proportion increases to more than one-third (38%) among patients with moderate or severe stroke [57]. It is therefore essential that pain assessment is a key focus of the initial diagnostic work-up and overall patient management. However, pain remains challenging to assess and manage due to its potentially multifactorial etiology and the interplay between different pathologies. A clinical algorithm for the differential diagnosis of shoulder pain mechanisms following CNS lesions is needed as effective pain management depends on distinguishing between neuropathic, nociceptive, and nociplastic pain [56]—a challenging task in clinical practice. However, despite these challenges, targeted interventions can provide effective pain relief. BoNT-A is a key therapeutic option in this area, with a positive impact on nociceptive pain reduction through the alleviation of muscle overactivity, as well as improvements in passive range of motion and quality of life in patients with shoulder pain after CNS lesions. For example, a systematic review and meta-analysis concluded that BoNT-A is effective at reducing pain and improving abduction and external rotation in patients with painful shoulder conditions following CNS lesions [31]. A randomized controlled trial in patients with pain arising from shoulder conditions following CNS lesions found that BoNT-A injected into the subscapularis muscle had a significant positive effect on pain, spasticity, passive external rotation without pain, and quality of life [32]. Further, a post hoc analysis of the TOWER study showed a correlation between improved proximal spasticity scores and pain scores when treatment for upper limb spasticity included injection into muscles acting on the shoulder [17]. There is even some evidence to suggest a positive effect of BoNT-A on central neuropathic pain, although data are currently limited [58]. Suprascapular nerve block also has a place in the management of shoulder pain, providing analgesia for up to several weeks; in this sense, its clinical utility is complementary to that of BoNT-A, the analgesic effects of which begin a few days after injection and persist for several months [33].

The proposed categorization of patients with CNS lesions and painful shoulder spasticity into six subgroups, based on time since lesion and other musculoskeletal involvement (Figure 2; Table 2), offers a practical framework for guiding management strategies. This classification may help clinicians target treatments toward specific clinical presentations (pain, spasticity, and functional limitations) according to recovery stage and musculoskeletal pathology.

As a final consideration, a key strength of the recommendations in this article is the expert panel’s extensive experience in spasticity and dystonia management (more than 150 years collectively). However, given the centrality of multidisciplinary care for patients with CNS lesions, further development of recommendations for the diagnosis and management of shoulder conditions in this patient population should involve a broader range of professional perspectives (including, for example, those of orthotists and therapists).

## 4. Conclusions

The objective of this article was to provide consensus-based guidance from an international panel of experts on best diagnostic and therapeutic practice in assessing and treating shoulder problems after acquired CNS lesions. The intention was to provide practical support for less experienced physicians and improve their confidence in managing this challenging and undertreated clinical problem. Expert recommendations are provided for the clinical assessment of patients with shoulder conditions after acquired CNS lesions, including spasticity, and for the categorization and treatment of those with pain. These recommendations have the potential to improve clinical practice, particularly in settings where clinicians may not be experienced in evaluating and treating patients with shoulder spasticity.

## 5. Materials and Methods

A two-day face-to-face meeting was held in Paris, France, on 30 and 31 March 2023. Eleven experts in shoulder conditions following acquired CNS lesions from Europe and Canada participated, collectively representing more than 150 years of experience in the management of spasticity and dystonia.

During the meeting, experts first discussed the identification, diagnosis, characterization, and treatment of shoulder conditions following CNS lesions. Second, they defined clear management strategies and pathways that account for clinically important variables (e.g., time since index event). This process was facilitated by expert presentations, review of three clinical cases demonstrating the experts’ approaches to assessment and treatment (including therapy outcomes), subsequent discussions, and two workshops. The experts were divided into two groups for participation in the workshops, which were held in parallel. One group discussed the clinical examination and investigation of shoulder spasticity, based on an anatomical approach and the presence of pain, and the second group discussed management strategies. The workshops were conducted in such a way that areas of consensus could be readily identified and treatment recommendations based on this consensus could be formulated. All experts who participated in the meeting are included as authors of the present article.

The meetings were audio-recorded. Concept drawings of the figures and tables were created during the workshops. Consolidated outcomes following the consensus meeting were extracted from the transcripts and used to form the basis of this article. Following the consensus process, 100% agreement between the expert panel on the topics discussed during the consensus-building process was achieved, and any disagreements were resolved during the consensus discussions. This resulted in the development of the included recommendations. Note that these recommendations are based on expert consensus without systematic literature review or empirical validation.

## Figures and Tables

**Table 1:** Recommended evaluation and diagnostic work-up of shoulder conditions in patients with acquired central nervous system lesions ^1^.

Diagnostic Component	Tools and Approaches
Patient history, including interpretation of reported symptoms	Assess: presence and type of pain; - nociceptive, neuropathic, and nociplastic. impact on function, activities, participation, and sleep; whether issues are activity dependent; care needs; problems with maintaining good hygiene, most importantly axillary hygiene.
Observation of postures and pain responses	Check presence and impact of: abnormal posture (pattern, at rest, during gait, during upper limb task); glenohumeral subluxation or shoulder subluxation; scapular position; muscle atrophy; skin lesions.
Clinical examination with targeted tests for specific signs	Examine: rotator cuff tendons ^2^ and ligaments; global and analytic ranges of motion in the affected joints; sensory evaluation (allodynia, hyperalgesia); muscle tone (stretching at slow/quick velocity in both passive and active shoulder movement), weakness/power, and contractures, related to: - rotator cuff muscles (teres minor, subscapularis, infraspinatus, supraspinatus); - scapular mobilizers and stabilizers (teres major, latissimus dorsi, serratus anterior, serratus posterior, trapezius, rhomboids, levator scapulae) [24]; - other muscular stabilizers (deltoids, pectoralis major and minor, biceps, triceps).
Diagnostic tests	Consider the following diagnostic tests: X-ray, ultrasound ± magnetic resonance imaging, and computed tomography; neurophysiology (nerve conduction studies [F- and H-waves], EMG, dynamic EMG, surface EMG polygraphy); nerve, muscle, and/or joint block (anesthetic block). Assess upper limb impairment using the Fugl–Meyer method [25]. Assess upper limb capacity with functional tests, for example: Box and Block test [26]; Action Research Arm Test [27]; ArmA.

^1^ This list is intended to cover all relevant tools and approaches that can be used to evaluate a patient presenting with upper limb spasticity, identify shoulder involvement, and guide tailored treatment. Not all these approaches will be needed for all patients. ^2^ Systematically evaluate for tendon pathology using appropriate provocation tests for the rotator cuff tendons, as guided by observation of pain with shoulder movements in abduction, flexion, external and internal rotation in neutral, and external rotation in abduction [28]. ArmA: Arm Activity Measure; EMG: electromyography.

**Table 2:** Treatment recommendations for the management of shoulder conditions following acquired central nervous system lesions ^1^.

Phase ^2^	Management Strategies to Consider
Acute	
Muscle overactivity and no other musculoskeletal involvement	Removal of triggering factors. Shoulder support. Sufficient range of motion for hygiene. Analgesics ^3^. Early mobilization, including physical/occupational therapy. Muscular electrical stimulation for muscle weakness. Transcutaneous electrical nerve stimulation for analgesia. BoNT-A (assess predictors for developing disabling spasticity).
Muscle overactivity with other musculoskeletal involvement	As above, plus: corticosteroid–local anesthetic infiltration for the following: - subacromial pathology; - long head of biceps tendonitis/tenosynovitis; - acromioclavicular joint arthritis; - glenohumeral joint arthritis; - capsulitis.
Post-acute	
Muscle overactivity and no other musculoskeletal involvement	BoNT-A. Suprascapular nerve block followed by BoNT-A. Physical/occupational therapy (as part of an interdisciplinary approach to treatment [37]). Shockwave therapy. Oral anti-spasticity agents (second-line).
Muscle overactivity with other musculoskeletal involvement	As above, plus: Corticosteroid–local anesthetic infiltration.
Chronic	
Muscle overactivity and no other musculoskeletal involvement	BoNT-A. Suprascapular nerve block followed by BoNT-A. Chemo- or thermoneurolysis. Surgery (neuro-orthopedic).
Muscle overactivity with other musculoskeletal involvement	As above, plus: Pulsed radiofrequency treatment. Cryoneurolysis. Chemoneurolysis (phenol, alcohol), if insufficient dose for BoNT-A. Needle tenotomies. No orthopedic surgery.

^1^ Treatment should always be patient centric, goal directed, and team based, and goals should be developed in collaboration with the patient and their family/caregiver [35,37]. ^2^ Acute: <2 weeks; post-acute: 2 weeks–6 months; chronic: >6 months [38]. ^3^ Early medical management (e.g., with gabapentinoids) should be considered whenever a neuropathic pain component is suspected. BoNT-A: botulinum neurotoxin type A.

## Data Availability

The original contributions presented in this study are included in the article. Further inquiries can be directed to the corresponding author.

## Abbreviations

The following abbreviations are used in this manuscript:

ArmA	Arm Activity Measure
BoNT-A	Botulinum neurotoxin type A
CNS	Central nervous system
CP	Cerebral palsy
EMG	Electromyography
GAS-eous	Goal Attainment Scaling-Evaluation of Outcomes for Upper Limb Spasticity
MAS	Modified Ashworth Scale
VAS	Visual Analog Scale
ViVe	Victoria Verona
